# Usage of Tranexamic Acid for Total Hip Arthroplasty: A Matched Cohort Analysis of 144,344 Patients

**DOI:** 10.3390/jcm13164920

**Published:** 2024-08-20

**Authors:** Anubhav Thapaliya, Mehul M. Mittal, Terrul L. Ratcliff, Varatharaj Mounasamy, Dane K. Wukich, Senthil N. Sambandam

**Affiliations:** 1 University of Texas Southwestern Medical School, 5323 Harry Hines Blvd, Dallas, TX 75390, USA; anubhav.thapaliya@utsouthwestern.edu (A.T.);; 2 Department of Orthopaedic Surgery, University of Texas Southwestern Medical Center, 1801 Inwood Road, Dallas, TX 75390, USA; terrul.ratcliff@utsouthwestern.edu (T.L.R.); orthovcu@gmail.com (V.M.); dane.wukich@utsouthwestern.edu (D.K.W.)

**Keywords:** tranexamic acid (TXA), total hip arthroplasty (THA), antifibrinolytics, perioperative surgery

## Abstract

**Background:** The literature is inconclusive regarding the potential complications of tranexamic acid (TXA), an antifibrinolytic drug, for total hip arthroplasty (THA). The purpose of this study is to compare complication rates and patient outcomes between THA patients administered TXA vs. THA patients not administered TXA. **Methods:** The TriNetX Research network was utilized to generate a cohort of adult patients who underwent THA between 2003 and 2024. These patients were categorized into two subgroups for the retrospective analysis: (1) patients who received TXA 24 h prior to THA (TXA), and (2) patients who did not receive TXA 24 h prior to total hip arthroplasty (no-TXA). The follow-up period was 30 and 90 days. **Results:** At 30 days following THA, the TXA patients had a reduced risk of transfusion (risk ratio (RR): 0.412; 95% confidence intervals (CI): 0.374, 0.453), reduced risk of DVT (RR: 0.856; CI: 0.768, 0.953), reduced risk of joint infection (RR: 0.808; CI: 0.710, 0.920), but a higher rate of periprosthetic fracture (RR: 1.234; CI: 1.065, 1.429) compared to patients who did not receive TXA. At 90 days following THA, TXA patients had a reduced risk of transfusion (RR: 0.446; CI: 0.408, 0.487), DVT (RR: 0.847; CI: 0.776, 0.924), and periprosthetic joint infection (RR: 0.894; CI: 0.815, 0.982) compared to patients who did not receive TXA. Patients who received TXA had higher rates of periprosthetic fracture (RR: 1.219; CI: 1.088, 1.365), acute postoperative anemia (RR: 1.222; CI: 1.171, 1.276), deep surgical site infection (SSI) (RR: 1.706; CI: 1.117, 2.605), and superficial SSI (RR: 1.950; CI: 1.567, 2.428) compared to patients who did not receive TXA. **Conclusions:** Patients receiving TXA prior to THA exhibited significantly reduced the prevalence of blood transfusions, DVT, and periprosthetic joint infection following THA. However, superficial SSI and periprosthetic fracture were seen with higher rates in the TXA cohort than in the no-TXA cohort.

## 1. Introduction

Tranexamic acid (TXA) is a synthetic drug, administered both topically and intravenously, that inhibits fibrinolysis and clot breakdown to reduce blood loss [1]. TXA can also be intravenously administered as a prophylactic measure to decrease blood loss and lower the likelihood of blood transfusion [2]. Moreover, TXA is typically administered within 24 h of a surgical procedure, as there is increased fibrinolytic activity in the first hours of surgery [3].

Total hip arthroplasty (THA) is a safe and routinely performed surgical intervention [4], with over 450,000 THA procedures performed annually in the United States (US) [5]. Primarily performed in the elderly, THA can treat the degenerative manifestations of end-stage hip joint disease as well as relieve hip joint pain and enhance the quality of life through improved joint mobility [6,7]. Projections indicate that by 2030, primary THA is expected to grow by 171%, with revision THA expected to increase by 142% in the same time frame [8]. However, despite the high prevalence of THA and 90% survivorship at 10 years [9], complications can result, ranging from periprosthetic dislocation and fractures to hematomas [10]. Postoperative complications are most common in elderly patient populations and comorbid patients [8,11], with common THA complications including blood loss and associated post-operative anemia [12]. Significant blood loss can necessitate allogenic blood transfusions, which introduces more potential complications and adverse events to the THA. Hence, research efforts are aimed at optimizing blood management to reduce transfusion rates and blood loss [13]. For example, Zhu et al. reported a statistically significant reduction in total blood loss, intraoperative blood loss, postoperative blood loss, hemoglobin drop, allogenic blood transfusion rate, and average hospital stay with TXA administration in THA when compared to controls [14]. 

Although the previous literature has established that TXA improves THA outcomes by reducing blood loss [15,16], the widespread adoption of TXA in the surgical community has been limited by possible side effects [3]. For example, due to its antifibrinolytic properties, TXA is regarded as an independent risk factor for venous thromboembolism [17]. Even in patients with low thrombotic risk, studies suggest a possible association between TXA administration and an increased risk of myocardial infarction (MI) [18]. Lower extremity deep vein thrombosis (DVT) is one of the more common complications following THA [19], with an incidence rate of 40–60% [20]. The development of DVT in the lower extremities can increase the risk of pulmonary embolism (PE) and other life-threatening complications as well as increase the hospital length of stay and treatment costs [19]. 

As outlined above, previous studies are inconclusive and present contradictory results regarding the specific complications and outcomes of TXA administration for THA. Moreover, there is a lack of a single study that examines multiple complications and patient outcomes via a large multicenter database. Considering this and the debate surrounding the efficacy of TXA, the purpose of this study is to compare complication rates and patient outcomes, at 30 days and 90 days post-procedure, between THA patients administered TXA vs. THA patients not administered TXA. This study is unique in that we will employ a large, nationally representative patient population. We hypothesize that patients administered TXA for THA will have fewer perioperative and postoperative complications compared to patients who were not administered TXA for THA.

## 2. Methods

### 2.1. Study Design and Data Source

The TriNetX Research network (https://trinetx.com, Baltimore, MD, USA) was utilized for this study. The TriNetX Research network features one of the largest repositories of data from the US, Canada, and Western Europe, encompassing inpatient, outpatient, and emergency visit data sourced from over 80 healthcare organizations (HCOs) and spanning more than 120 million patient records. Furthermore, patient data are enriched with information from over 100 commercial and government payers, including Medicare [21,22].

Patients 18 years old and above who underwent THA between 1 January 2003 and 1 January 2024 met the inclusion criteria. The data were sourced via the TriNetX database on 18 April 2024. These patients were categorized into two cohorts: (1) patients who received TXA 24 h prior to total hip arthroplasty (TXA), and (2) patients who did not receive TXA 24 h prior to total hip arthroplasty (no-TXA). The selection of patients utilized appropriate CPT, ICD-9, and ICD-10 codes. Further details on cohort construction can be found in the Supplementary Materials File.

### 2.2. Index Event and Outcome Analysis

The study assessed common perioperative and postoperative complications, which are further elaborated on in the Section 3. The index event was defined as the initiation of analysis for each patient, which, in this study, corresponds to the date of THA for each particular patient. The follow-up period for this study was 30 and 90 days (97% follow-up rate). Additional information on the index event, outcomes of interest, and follow-up duration is provided in the Supplementary Materials File.

### 2.3. Statistical Tools, Data Analysis, and Propensity Score Matching

The relative risk, supplemented by absolute risk, was used to compare the risk of complications between the exposure and comparison groups, with 95% confidence intervals provided for all relative risk calculations. The statistical tests used included Fisher’s exact test and Chi-square for categorical variables, and Student’s *t*-test for continuous variables. *p* values < 0.01 were considered significant.

Patients in the TXA and no-TXA cohorts were subjected to matching based on age, sex, smoking status, diabetes, and overweight/obesity status using a greedy nearest neighbor matching algorithm. Standard mean differences were analyzed to ensure balance between the cohorts after matching. The before and after matching data are presented in Table 1, and the matched characteristics can be found in the Supplementary Materials File.

### 2.4. Software Used for Statistical Analysis, Validation, and Data Visualization

The TriNetX Live platform was used for data compilation. Microsoft Excel (2023) was utilized for further analysis and data visualization. The analytical procedures were verified independently by all co-authors and further confirmed by the corresponding author (SS).

### 2.5. Data Integrity and Ethical Considerations

All information within the TriNetX database is compliant with the Health Insurance Portability and Accountability Act (HIPAA) and contains only de-identified aggregate information [23]. As a result, this study was exempt from Institutional Review Board (IRB) approval by UT Southwestern IRB.

## 3. Results

### 3.1. Patient Demographic Data Analysis

A total of 180,149 patients were identified via the TriNetX database as having undergone THA during the specified time frame, including 107,912 patients in the TXA cohort (59.90%) and 72,237 patients in the no-TXA cohort (40.10%). The average age at the time of THA was significantly higher for the TXA patient cohort (64.4 ± 11.1) as compared to the no-TXA patient cohort (63.4 ± 11.6) (*p* < 0.001). For both the TXA and no-TXA cohorts, there was a greater proportion of female patients than male patients with 54,159 (50%) females and 44,129 (41%) males in the TXA cohort and 37,747 (52%) females and 33,476 (46%) males in the no-TXA cohort (Table 1). After propensity matching, 72,172 TXA patients and 72,172 no-TXA patients were included in the analysis. After matching, 52% of the cohort in each group were females and 46% were males (*p* < 0.001), and there were no differences in the rates of diabetes mellitus or obesity. The frequency of racial and ethnicity groups between TXA and no-TXA patients was Caucasian (82%, 80%), Black/African American (9%, 10%), Hispanic/Latino (3%, 4%), Other (2%, 2%), and Asian (1%, 1%), respectively (Table 2).

### 3.2. Analysis of Patient Complications 

Thirty-Day Follow-Up:

At 30 days following THA, TXA patients had a reduced risk of transfusion (risk ratio (RR): 0.412; 95% confidence interval (CI): 0.374, 0.453), reduced risk of DVT (RR: 0.856; CI: 0.768, 0.953), reduced risk of periprosthetic joint infection (RR: 0.808; CI: 0.710, 0.920), but a higher rate of periprosthetic fracture (RR: 1.234; CI: 1.065, 1.429) compared to patients who did not receive TXA. There were no significant differences between the two groups regarding the rates of MI, PE, hematoma formation, acute renal failure, wound dehiscence, pneumonia, deep or superficial surgical site infection (SSI), and periprosthetic dislocation (Table 3).

Ninety-Day Follow-Up:

At 90 days following THA, TXA patients had a reduced risk of transfusion (RR: 0.446; CI: 0.408, 0.487), DVT (RR: 0.847; CI: 0.776, 0.924), and periprosthetic joint infection (RR: 0.894; CI: 0.815, 0.982) compared to patients who did not receive TXA. Patients who received TXA had higher rates of periprosthetic fracture (RR: 1.219; CI: 1.088, 1.365), acute postoperative anemia (RR: 1.222; CI: 1.171, 1.276), deep SSI (RR: 1.706; CI: 1.117, 2.605), and superficial SSI (RR: 1.950; CI: 1.567, 2.428) compared to patients who did not receive TXA. There were no significant differences between the two groups at 90 days regarding the rates of MI, hematoma formation, acute renal failure, pneumonia, wound dehiscence, and prosthetic-related dislocations and mechanical complications (Table 4).

## 4. Discussion

The utilization of THA continues to increase, especially with the increasing average patient age and consequent higher prevalence of degenerative hip diseases [4,24]. The indications for the surgical administration of TXA, as a cost-effective measure to minimize blood loss, are also continuing to expand [25,26]. However, the thrombotic and life-threatening cardiovascular complications associated with TXA administration has limited widespread employment of TXA [3,17,18,27]. 

Patients in the no-TXA cohort had a greater need for blood transfusions at both the 30-day and 90-day follow-ups compared to the patients in the TXA cohort. Given that TXA serves an antifibrinolytic role and minimizes blood loss [13,28,29], this finding is consistent with our hypothesis. Our analysis is supported by a study by Stoicea et al. which found that TXA, via both intravenous and intra-articular forms, reduced decreases in both postoperative hemoglobin and hematocrit following primary posterior and revision THA, indicating the efficacy of TXA in minimizing blood loss and transfusion [3]. Other studies and reviews also align with our finding that TXA promotes hemostasis and reduces intraoperative blood loss in orthopedic procedures [2,12,30]. 

Patients in the TXA cohort had a significantly reduced risk of DVT and need for transfusion compared to patients in the no-TXA cohort at both 30 and 90 days. These results are congruent with most of the previous orthopedic literature, apart from the findings of TXA reduction in the rates of DVT. Two separate meta-analyses of randomized controlled trials, investigating the efficacy and safety of TXA in orthopedic lower limb surgeries, reported that TXA did not increase the risk of venous thromboembolism, and it reduced blood loss, transfusion requirements, and length of hospital stay without any additional thromboembolic risk [14,31]. Other studies reported similar findings that the use of high-dose TXA does not influence the prevalence of MI, DVT, and PE [3,32,33,34]. Although some studies have found correlations between TXA and MI [18,27,34], these studies primarily studied patients at high-risk for cardiovascular disease. We did not observe any difference in the rate of postoperative MI between the two groups. 

An interesting observation was that patients who received TXA had higher rates of superficial SSI at 30 days and higher rates of both superficial and deep infection as well as prosthetic fracture at 90 days. These findings are not concordant with the current consensus that TXA is not known to be associated with increasing rates of SSI. A meta-analysis reviewed 31 articles and concluded that the intravenous administration of TXA reduces the incidence of overall infection, including SSI, in patients undergoing both THA and TKA [35]. One hypothesized mechanism for this decrease in infection rates is TXA-associated changes in immune marker expression on immune cell subsets and decreased levels of proinflammatory cytokines, both of which are correlated with a lower rate of postoperative infection [36]. Moreover, blood transfusion is a known risk for prosthetic joint infection [35], but the TXA cohort had decreased rates of blood transfusion. We do not have a good explanation for this finding since the rate of postoperative hematoma formation was not different between the two groups. Kramer et al. found that there is no added risk for wound healing problems or SSI attributable to preoperative TXA in spine surgeries [37]. This discrepancy can be explained by patient demographics: a large proportion of the patients undergoing THA are geriatric [4], a patient population that presents with comorbidities and at higher risk for orthopedic and non-orthopedic complications, such as SSI and periprosthetic fracture [38]. The lack of difference in hematoma complications between TXA and no-TXA groups are in congruence with the previous literature given that TXA mechanistically works to prevent hematomas [39]. 

Patients undergoing THA who were given preoperative TXA had lower rates of periprosthetic joint infection at the 30- and 90-day follow-ups compared to patients who did not receive preoperative TXA. These findings are consistent with the previous literature. Yazdi et al. studied TXA in primary joint arthroplasty and reported that TXA helps to reduce the rate of periprosthetic joint infection. The authors proposed that a reduction in bleeding and a lower need for allogenic blood transfusion may be responsible for the lower incidence of infection [40]. Another study, upon adjusting for multiple patient characteristics and surgical factors, independently associated TXA with a reduced risk of subsequent acute periprosthetic joint infection [41]. Additionally, other researchers concluded that TXA has inhibitory effects against implant infections by reducing surgical site bleeding and associated biofilm formation [42]. Regarding acute renal failure, although there is research in the literature suggesting a potential renal effect of TXA and discouraging administration of TXA to patients with kidney dysfunction [29], we found no significant differences in kidney failure between TXA and no-TXA cohorts.

A statistically significant difference in wound dehiscence was not observed at the 30-day follow-up. Several studies have reported that TXA did not add risk to wound healing complications in orthopedic procedures [37,43]. Our study agrees with the literature regarding the negligible effect of TXA on wound healing complications.

Our study’s inherent limitations stem from its retrospective nature and the fact that patient data were sourced from EHRs which are susceptible to errors in coding and documentation. The TriNetX database is a voluntary program, and selection bias may be present due to overrepresentation by large, academic research institutions. TriNetX provides temporality on a day-to-day level, and the route of administration and dosage information was lacking, and therefore, it was excluded from the analysis. Hence, TriNetX cannot provide more specific timing information. Furthermore, the data may not be representative of the entire global population. We could not adjust our outcomes of interest after THA for socioeconomic variables such health insurance, education level, and income, and we acknowledge that these variables are important to consider, particularly in any study that outcomes after surgery. 

Since the patient data were derived from a diverse range of medical practices and locations across the US, Western Europe, and Canada, there were likely variations in surgical equipment, surgical techniques, reporting of medical and surgical complications, and post-operative patient protocol. For example, the specific type of periprosthetic joint infection and its exact treatment course is not known for each individual patient. Moreover, retrospective studies are reliant on the medical personnel to record patient data. Any inaccuracies in data entry or reporting may have influenced the results and analysis of our study. However, the size of our patient cohorts mitigates these risks and enhances the predictive power of the study, allowing for a more accurate estimate of rare complications. Additionally, all complications of interest were decided upon prior to collecting data, which solidifies the reliability of the study. Regarding biases, because none of the study’s authors participated in the actual surgical procedures and patients were de-identified, the possibility of bias was reduced, further enhancing the reliability of our findings. 

Despite the limitations described, a particular strength of this study is the ability to analyze a large, matched cohort of patients who underwent THA, incorporating important demographic factors and comorbidities. To the best of our knowledge, this is the largest known patient cohort to be used in comparing THA patient outcomes in patients who received or did not receive preoperative TXA. Additionally, this study is strengthened by its use of propensity score matching, a validated observational cohort comparison technique, which helps remove any confounding bias [44]. Propensity score matching allows for factors such as age, sex, obesity status, and tobacco use from confounding the result outputs. We recognize that 90 days is a relatively short period of follow-up, but this should be adequate to address the potential complications of TXA following THA. Given that many hospital metrics are based on 90-day mortality, longer follow-up periods may also alter measurements and change the significance of the findings. 

## 5. Conclusions

At 30 days following THA, TXA patients had a reduced risk of transfusion, reduced risk of DVT, reduced risk of prosthetic joint infection, but a higher rate of periprosthetic fracture compared to patients who did not receive TXA. At 90 days following THA, TXA patients had a reduced risk of transfusion, lower extremity DVT, and prosthetic joint infection compared to patients who did not receive TXA. At 90 days following THA, patients who received TXA had higher rates of postoperative anemia, deep SSI, and superficial SSI compared to patients who did not receive TXA. No significant differences in the rates of postoperative MI were observed between the two groups at either the 30- or 90-day follow-up following THA. Future studies should examine the complications of TXA with other arthroplasty procedures and aim for long-term follow-up and with a large, representative patient population.

## Figures and Tables

**Table 1:** Patient demographic characteristics before match.

Patient Demographic Characteristics (Before Match)			
	TXA (107,912)	No-TXA (72,237)	
Characteristic	*n* (Mean or %)	*n* (Mean or %)	*p*
Age at Index	107,912 (64.4 ± 11.1)	72,237 (63.4 ± 11.6)	<0.001
Sex			
Male	44,129 (41%)	33,476 (46%)	<0.001
Female	54,159 (50%)	37,747 (52%)	<0.001
Race and Ethnicity			
Hispanic or Latino	2501 (2%)	2754 (4%)	<0.001
Asian	1075 (1%)	537 (1%)	<0.001
Black or African American	8535 (8%)	7279 (10%)	<0.001
White	81,573 (76%)	57,752 (80%)	<0.001
Other Race	1544 (1%)	1251 (2%)	<0.001
Diagnosis			
Tobacco Use	3455 (3%)	1532 (2%)	<0.001
Diabetes Mellitus	14,865 (14%)	9260 (13%)	<0.001
BMI			
At Most 18.5 kg/m^2^	1375 (1%)	1078 (1%)	<0.001
18.5–25 kg/m^2^	10,068 (9%)	7148 (10%)	<0.001
25–30 kg/m^2^	17,190 (16%)	12,220 (17%)	<0.001
30–35 kg/m^2^	14,334 (13%)	10,546 (15%)	<0.001
35–40 kg/m^2^	8309 (8%)	6098 (8%)	<0.001
At Least 40 kg/m^2^	4377 (4%)	3142 (4%)	0.002

**Table 2:** Patient demographic characteristics after match.

Patient Demographic Characteristics (After Match)			
	TXA (72,172)	No-TXA (72,172)	
Characteristic	*n* (Mean or %)	*n* (Mean or %)	*p*
Age at Index	72,172 (63.4 ± 11.6)	72,172 (63.4 ± 11.6)	0.5385
Sex			
Male	33,397 (46%)	33,425 (46%)	0.883
Female	37,761 (52%)	37,733 (52%)	0.883
Race and Ethnicity			
Hispanic or Latino	1897 (3%)	2743 (4%)	<0.001
Asian	744 (1%)	535 (1%)	<0.001
Black or African American	6226 (9%)	7265 (10%)	<0.001
White	58,896 (82%)	57,712 (80%)	<0.001
Other Race	1166 (2%)	1247 (2%)	0.096
Diagnosis			
Tobacco Use	1487 (2%)	1532 (2%)	0.408
Diabetes Mellitus	9081 (13%)	9260 (13%)	0.157
BMI			
At Most 18.5 kg/m^2^	951 (1%)	1055 (1%)	0.019
18.5–25 kg/m^2^	6904 (10%)	7120 (10%)	0.055
25–30 kg/m^2^	12,085 (17%)	12,207 (17%)	0.391
30–35 kg/m^2^	10,516 (15%)	10,537 (15%)	0.876
35–40 kg/m^2^	6002 (8%)	6096 (8%)	0.372
At Least 40 kg/m^2^	3075 (4%)	3142 (4%)	0.385

**Table 3:** Table of risk ratios (30-day follow-up—matched).

Table of Risk Ratios—30-Day F/U (Matched)							
Measure	TXA (*n*)	No-TXA (*n*)	TXA Proportion	No-TXA Proportion	Risk Ratio	95% CI	*p*
Transfusion	593	1440	0.8%	2.0%	0.412	(0.374, 0.453)	<0.001
Myocardial Infarction	271	267	0.4%	0.4%	1.015	(0.857, 1.201)	0.863
Pulmonary Embolism	373	412	0.5%	0.6%	0.905	(0.787, 1.041)	0.163
Deep Vein Thrombosis (Lower Extremity)	605	707	0.8%	1.0%	0.856	(0.768, 0.953)	0.005
Hematoma	39	34	0.1%	0.0%	1.147	(0.724, 1.817)	0.558
Periprosthetic Joint Infection	409	506	0.6%	0.7%	0.808	(0.710, 0.920)	0.001
Acute Renal Failure	939	1018	1.3%	1.4%	0.922	(0.845, 1.007)	0.072
Acute Posthemorrhagic Anemia	3796	3129	5.3%	4.3%	1.213	(1.158, 1.271)	<0.001
Wound Dehiscence	312	291	0.4%	0.4%	1.072	(0.914, 1.257)	0.391
Pneumonia	351	404	0.5%	0.6%	0.869	(0.753, 1.002)	0.053
Deep SSI	26	18	0.0%	0.0%	1.444	(0.792, 2.634)	0.228
Superficial SSI	107	55	0.1%	0.1%	1.945	(1.406, 2.693)	<0.001
Periprosthetic Mechanical Complication	75	99	0.1%	0.1%	0.758	(0.561, 1.022)	0.069
Periprosthetic Dislocation	330	357	0.5%	0.5%	0.924	(0.796, 1.073)	0.302
Periprosthetic Fracture	396	321	0.5%	0.4%	1.234	(1.065, 1.429)	0.005
Subgroup Analysis							
Myocardial Infarction (Previous Stent/CABG vs. No Previous Stent/CABG)	33	21	4.3%	2.7%	1.571	(0.918, 2.691)	0.097

**Table 4:** Table of risk ratios (90-day follow-up—matched).

Table of Risk Ratios—90-Day F/U (Matched)							
Measure	TXA (N)	No-TXA (*n*)	TXA Proportion	No-TXA Proportion	Risk Ratio	95% CI	*p*
Transfusion	713	1599	1.0%	2.2%	0.446	(0.408, 0.487)	<0.001
Myocardial Infarction	385	402	0.5%	0.6%	0.958	(0.833, 1.101)	0.543
Pulmonary Embolism	565	634	0.8%	0.9%	0.891	(0.796, 0.998)	0.045
Deep Vein Thrombosis (Lower Extremity)	920	1086	1.3%	1.5%	0.847	(0.776, 0.924)	<0.001
Hematoma	58	48	0.1%	0.1%	1.208	(0.824, 1.771)	0.331
Periprosthetic Joint Infection	830	928	1.2%	1.3%	0.894	(0.815, 0.982)	0.019
Acute Renal Failure	1298	1328	1.8%	1.8%	0.977	(0.906, 1.054)	0.555
Acute Posthemorrhagic Anemia	4415	3612	6.1%	5.0%	1.222	(1.171, 1.276)	<0.001
Wound Dehiscence	626	552	0.9%	0.8%	1.134	(1.012, 1.271)	0.030
Pneumonia	568	614	0.8%	0.9%	0.925	(0.826, 1.036)	0.179
Deep SSI	58	34	0.1%	0.0%	1.706	(1.117, 2.605)	0.012
Superficial SSI	236	121	0.3%	0.2%	1.950	(1.567, 2.428)	<0.001
Periprosthetic Mechanical Complication	191	196	0.3%	0.3%	0.974	(0.799, 1.189)	0.799
Periprosthetic Dislocation	603	647	0.8%	0.9%	0.932	(0.835, 1.041)	0.211
Periprosthetic Fracture	658	540	0.9%	0.7%	1.219	(1.088, 1.365)	0.001
Subgroup Analysis							
Myocardial Infarction (Previous Stent/CABG vs. No Previous Stent/CABG)	46	35	5.9%	4.5%	1.314	(0.857, 2.017)	0.209

## Data Availability

The data that support the findings of this study are available from TriNetX. Restrictions apply to the availability of these data, which were used under license for this study. Data are available from https://trinetx.com with the permission from TriNetX.

## Supplementary Materials

The following supporting information can be downloaded at: https://www.mdpi.com/article/10.3390/jcm13164920/s1. Supplementary Materials File.
