# Supplementary material for: Usage of Tranexamic Acid for Total Hip Arthroplasty: A Matched Cohort Analysis of 144,344 Patients
Source: J Clin Med. 2024 Aug 20;13(16):4920. doi: 10.3390/jcm13164920 (PMC11355791; doi:10.3390/jcm13164920)
Supplement: Supplementary file 1 [file jcm-13-04920-s001.zip › jcm-3128561-supplementary.pdf]

Supplementary File

A – Cohort Selection
Terms & Definitions

Cohort 1: No TXA Hip Arthroplasty

1) Inclusion
- Age ≥ 18
- Hip Arthroplasty (see "Other Coded Variables")
- *Subgroup analysis:*
    - *Coronary artery bypass graft (CABG) or intracoronary stent (see "Other Coded Variables")*

2) Exclusion
- Tranexamic Acid

Cohort 2: TXA Hip Arthroplasty

1) Inclusion
- Age ≥ 18
- Hip Arthroplasty (see "Other Coded Variables")
- Tranexamic Acid
- *Subgroup analysis:*
    - *Coronary artery bypass graft (CABG) or intracoronary stent (see "Other Coded Variables")*

B – Index Setup
Timing

The index event for all cohorts is day of Hip Arthropaslty surgery (see "Other Coded Variables")

C – Count & Follow-up
Timing

Propensity score matching was performed on age at index event, sex, Tobacco Use (see "Other Coded Variables") and Overweight and Obesity (see "Other Coded Variables").

Follow-up table:

30 Days	Approx. 99%
90 Days	Approx. 97%

Range & Region:
Data encompasses 2003-2024, Research Network

D – Outcomes & Composites
Multi-code Variables & Other Codes

Outcome Variables

- Transfusion
    - Transfusion of Nonautologous Red Blood Cells into Peripheral Vein, Percutaneous Approach (UMLS:ICD10PCS:30233N1).

- Myocardial Infarction
    - Acute myocardial infarction (UMLS:ICD10CM:I21).

- Pulmonary Embolism
    - Pulmonary embolism (UMLS:ICD10CM:I26).

- Deep Vein Thrombosis (Lower Extremity)
    - Acute embolism and thrombosis of deep veins of lower extremity (UMLS:ICD10CM:I82.4).

- Hematoma
    - Nontraumatic hematoma of soft tissue (UMLS:ICD10CM:M79.81).

- Periprosthetic Joint Infection
    - Infection and inflammatory reaction due to internal joint prosthesis (UMLS:ICD10CM:T84.5).

- Acute Renal Failure
    - Acute kidney failure (UMLS:ICD10CM:N17).

- Acute Posthemorrhagic Anemia
    - Acute posthemorrhagic anemia (UMLS:ICD10CM:D62).

- Wound Dehiscence
    - Disruption of wound, unspecified, initial encounter (UMLS:ICD10CM:T81.30XA); or
    - Disruption of external operation (surgical) wound, not elsewhere classified, initial encounter (UMLS:ICD10CM:T81.31XA); or
    - Disruption of internal operation (surgical) wound, not elsewhere classified, initial encounter (UMLS:ICD10CM:T81.32XA).

- Pneumonia
    - Unspecified acute lower respiratory infection (UMLS:ICD10CM:J22); or
    - Unspecified bacterial pneumonia (UMLS:ICD10CM:J15.9); or
    - Pneumonia, unspecified organism (UMLS:ICD10CM:J18.9).

- Deep SSI
    - Infection following a procedure, deep incisional surgical site (UMLS:ICD10CM:T81.42).

- Superficial SSI
    - Infection following a procedure, superficial incisional surgical site (UMLS:ICD10CM:T81.41).

- Periprosthetic Mechanical Complication
    - Other mechanical complication of internal joint prosthesis (UMLS:ICD10CM:T84.09).

- Periprosthetic Dislocation
    - Dislocation of internal joint prosthesis (UMLS:ICD10CM:T84.02).

- Periprosthetic Fracture
    - Broken internal joint prosthesis (UMLS:ICD10CM:T84.01); or
    - Fracture of bone following insertion of orthopedic implant, joint prosthesis, or bone plate (UMLS:ICD10CM:M96.6); or
    - Periprosthetic fracture around internal prosthetic hip joint (UMLS:ICD10CM:M97.0).

Other Coded Variables

- Hip Arthroplasty
    - CPT:27130 – Arthroplasty, acetabular and proximal femoral prosthetic replacement (total hip arthroplasty), with or without autograft or allograft

- Tranexamic Acid
    - NLM:RXNORM:10691

- Tobacco Use
    - USMLS:ICD10CM:Z72.0

- Overweight and Obesity
    - USMLS:ICD10CM:E66
- CABG
    - CPT:1006199
    - CPT:1006207

- CPT:1006216
- CPT:1021165
